# Allopathy Versus Homœopathy

**Published:** 1848-04

**Authors:** 


### ALLOPATHY VERSUS HOMOEOPATHY.

To argue that the hundred-millionth part of a thousand-billionth of a grain of one of our common medicines can exert *no* influence on the living body, seems much like attempting to prove a negative, or vanquish a ghost with sword and pistols; yet that is the enterprise in which our esteemed colleague, Professor Miller, was engaged, when he delivered, before the Medical Society of Louisville, a discourse, of which its Secretary has sent us a copy. What it was which provoked that soberminded and deep-thinking Professor into this question, we do not even venture to guess. Whatever it might have been, it was an active stimulant, for his arguments and sarcasms were not dealt out in homœopathic doses. We fear, however, that it will turn out that all his allopathy has failed to cure the disease. Its roots, like those of a cancer, are too deeply infixed in the human mind to be eradicated, however sturdy the arm which wields the mattock. We ought rather to say, that the diathesis of ignorance and credulity, like the cancerous, will reproduce the disorder, in spite of all excision, and that until *it* shall be corrected, there can be no cure. Meanwhile, it is quite as well to administer argument and reproof in homœopathic doses—and those few and far between.

The sum and substance of all beneficial homœopathic practice may be comprised under three heads: 1, the disuse of medicines when their action is injurious; 2, abridgement of diet; 3, the use of active medicines by *physicians* who declaim against them, and *profess* to have laid them aside for infinitesimal portions; all of which benefits might be attained without resorting to the ridiculous visions of Hahnemann. But lest we should violate our own canon by transcending a homœopathic dose, we shall dismiss the subject.

